# Periodontitis was associated with worse clinical outcomes after catheter ablation for paroxysmal atrial fibrillation

**DOI:** 10.3389/fcvm.2022.1061243

**Published:** 2023-01-09

**Authors:** Akira Tashiro, Taishi Yonetsu, Norio Aoyama, Yuka Shiheido-Watanabe, Takayuki Niida, Shinsuke Miyazaki, Yasuhiro Maejima, Masahiko Goya, Mitsuaki Isobe, Takanori Iwata, Tetsuo Sasano

**Affiliations:** ^1^Department of Cardiovascular Medicine, Tokyo Medical and Dental University, Tokyo, Japan; ^2^Department of Periodontology, Kanagawa Dental University, Yokosuka, Japan; ^3^Sakakibara Heart Institute, Tokyo, Japan; ^4^Department of Periodontology, Graduate School of Medical and Dental Sciences, Tokyo Medical and Dental University, Tokyo, Japan

**Keywords:** catheter ablation, atrial fibrillation, paroxysmal atrial fibrillation (PAF), arrhythmia recurrence, periodontitis (PD), oral health status

## Abstract

**Background:**

Periodontitis (PD), a common chronic inflammatory disease, may be associated with the subsequent development of atrial fibrillation (AF) through a mechanism of systemic inflammation. However, little is known about the impact of PD on the recurrence of atrial fibrillation after catheter ablation (CA).

**Methods:**

A total of 132 patients (age 62.2 ± 10.6 years; 72.7% male) who underwent periodontal examinations and the first CA for paroxysmal atrial fibrillation (PAF) were investigated. Clinical periodontal examination was performed by independent trained periodontists, and patients were diagnosed with PD when the maximum periodontal probing depth was equal to or greater than 4 mm and bleeding on probing was evident. Of these, 71 patients (54%) were categorized as those with PD (PD group) and the other 61 (46%) as those without PD (non-PD group). Pulmonary vein isolation was performed in a standard fashion.

**Results:**

Kaplan–Meier curve analysis revealed worse atrial arrhythmia recurrence-free survival probabilities after CA for PAF in the PD group than in the non-PD group (64.8% versus 80.3%, respectively; *p* = 0.024) during a median follow-up period of 3.0 (interquartile range: 1.1–6.4) years. Cox regression analysis revealed PD as a significant predictor of arrhythmia recurrence (hazard ratio: 2.063, 95% confidence interval: 1.018–4.182), after adjusting for age and gender.

**Conclusion:**

Periodontitis was independently associated with an increased risk of arrhythmia recurrence after the first CA for PAF. Our results may suggest that the periodontal status is potentially a modifiable determinant of the outcomes after PAF ablation, and further prospective studies are warranted.

## 1. Introduction

Periodontitis (PD) is a chronic inflammatory disease primarily initiated by the response to periodontopathic bacteria in the periodontium surrounding and supporting teeth (1). The global burden of severe PD was estimated as 1.1 billion cases worldwide in 2019 (2). Previous epidemiological studies have suggested that PD may be a potential risk factor of a variety of heart diseases, including coronary artery disease (3–5), and atrial fibrillation (AF) (6). AF is the most common type of sustained cardiac arrhythmia in adults, is widely recognized as one of the main causes of stroke and poses a significant burden to patients and to public health systems (7). Paroxysmal AF (PAF) is a subtype of AF defined by a spontaneous or intervened termination within 7 days from onset, which is often targeted by the rhythm control strategy using anti-arrhythmic drugs or catheter ablation (CA). Despite significant advancement in CA technology, the non-negligible proportion of patients with PAF still had arrhythmia recurrence after the procedure (8). Previous studies have suggested various risk factors of recurrent AF after CA, including anatomical factors, comorbidities, and biomarkers. Notably, the inflammatory status, represented by higher levels of inflammatory biomarkers, has been reported to be relevant to the recurrence of arrhythmia (9). As a manifestation of chronic inflammation inducing recurrent arrhythmia, PD was investigated in previous studies (6, 10, 11). A recent study has shown the association between the serum antibodies to the periodontal pathogens and the recurrence of AF (11). Nevertheless, the impact of PD that is clinically diagnosed by periodontal examinations assessing clinical outcomes after AF ablation has not been elucidated. The present study sought to investigate the association between baseline PD evaluated at the time of the first CA for PAF and subsequent recurrence rate after CA.

## 2. Materials and methods

### 2.1. Study population

The prospective observational registry for the assessment of the association between cardiovascular disease and periodontal disease was conducted between May 2012 and August 2015 at Tokyo Medical and Dental University Hospital. The registry enrolled 1,000 consecutive patients with written informed consent who were admitted to the Department of Cardiovascular Medicine. The original protocol was approved in March 2012 by the institutional review board (IRB) of the Tokyo Medical and Dental University (MD2000-1165). All participants underwent a periodontal examination during hospitalization to assess their periodontal status. The ancillary protocol, which was additionally approved by the IRB in 2020 (M2020-020), was performed to collect the long-term clinical follow-up data of the participants from the original protocol by reviewing the clinical records in 2020. Of the 1,000 enrolled patients, a total of 135 patients who were hospitalized to undergo first CA for PAF were identified. After excluding three patients in whom the periodontal status was not examined (insufficient number of teeth), 132 patients were investigated in the present study. This study complies with the ethical principles of the Declaration of Helsinki.

### 2.2. Clinical periodontal examination

Periodontal examinations were performed before CA by three independent periodontists (certified by the Japanese Society of Periodontology) who were blinded to the patients’ characteristics and cardiovascular disease statuses. The number of remaining teeth were counted. The probing pocket depth (PPD), clinical attachment level (CAL), bleeding on probing (BoP), and Community Periodontal Index (CPI) at six points per tooth (buccal-mesial, mid-buccal, buccal-distal, lingual-mesial, mid-lingual, and lingual-distal) on six representative teeth (an upper right molar, an upper incisor, an upper left molar, a lower right molar, a lower incisor, and a lower left molar) were measured using a manual probe (PCP-UNC 15, Hu-Friedy, Chicago, IL, USA). When the corresponding tooth was missing, the adjacent tooth was used instead. PPD was defined as the distance from the gingival margin to the bottom of the periodontal pocket, CAL was defined as the distance from the cementoenamel junction to the bottom of the periodontal pocket, and BoP was defined as bleeding from the gingiva at the probe tip. CPI is a screening measurement of the periodontal condition that assesses the presence or absence of periodontal pockets, calculus, and gingival bleeding, and it is scored from 0 to 4. In this study, patients were diagnosed with PD when the maximum PPD was equal to or greater than 4 mm with positive BoP.

### 2.3. Examination of periodontopathic bacteria

The presence of three major periodontal bacteria (*Porphyromonas gingivalis*, *Prevotella intermedia*, and *Aggregatibacter actinomycetemcomitans*) in the periodontal pocket and saliva were evaluated using real-time polymerase chain reaction. Furthermore, the serum immunoglobulin G (IgG) titer against each bacterium was measured.

### 2.4. Baseline patients’ characteristics

The patient’s medical history, medications, smoking history, and alcohol consumption were recorded; physical examination was performed on admission. Peripheral blood samples were obtained for a blood cell count and the concentrations of albumin, creatinine, total cholesterol, triglyceride, high-density lipoprotein cholesterol, low-density lipoprotein cholesterol, hemoglobin A1c, C-reactive protein (CRP), and brain natriuretic peptide (BNP). The left ventricular ejection fraction and left atrial diameter were evaluated by transthoracic echocardiography before CA.

### 2.5. Ablation procedure

All procedures were performed under moderate or deep sedation and continuous heparinization to maintain the activated clotting time >300 s after the transseptal puncture. All patients in the current study underwent pulmonary vein (PV) isolation with a radiofrequency catheter under the guidance of a three-dimensional mapping system (CARTO-3; Biosense Webster, Diamond Bar, CA, USA), cryoablation (Arctic Front Advance; Medtronic Inc., Minneapolis, MN, USA), or hot balloon (SATAKE HotBalloon; Toray Industries, Inc., Tokyo, Japan) at the operator’s discretion. The procedural endpoint was defined as the electrical isolation of the PV. Additionally, a majority of the patients underwent cavotricuspid isthmus ablation, and some patients underwent adjunctive ablation (linear and focal ablation) at the operator’s discretion.

### 2.6. Clinical outcomes

Clinical outcomes after CA were assessed by reviewing the medical records. Atrial arrhythmia recurrence was defined as an episode of AF, atrial flutter, or atrial tachycardia lasting 30 s or longer after a 3-month blanking period, with or without the use of antiarrhythmic drugs. 12-lead electrocardiogram was recorded at every visit to the outpatient clinic, and 24-h Holter monitoring or 1-week event loop recorder was encouraged to be performed at 3- and 6-months and every 6-month thereafter. If patients were symptomatic suggesting atrial arrhythmia recurrence, an electrocardiogram, Holter monitoring, or event loop recorder was performed in addition.

### 2.7. Statistical analysis

All statistical analyses were performed using R software (version 4.0.3; R Foundation for Statistical Computing, Vienna, Austria). Continuous variables are presented as mean ± standard deviation or median with interquartile range (25th–75th percentile) and analyzed using the Student’s *t*-test or Mann–Whitney U test, as appropriate. Categorical variables were presented as numbers with percentages and analyzed using the chi-squared test or Fisher’s exact test, as appropriate. Kaplan–Meier analysis was performed to compare clinical outcomes between the patients with and without PD. To provide separate descriptions of the short- and long-term risks of recurrences, a landmark analysis with a landmark set at 1 year was performed. Cox proportional hazards model was used to identify the independent predictors of recurrent arrhythmia after CA. The associated variables showing a *p*-value < 0.150 in univariate analysis were entered in the multivariate model with patients’ age and gender, and stepwise regression was performed using the Akaike information criterion to fit the regression model.

## 3. Results

### 3.1. Patient characteristics

Of the 132 patients, 71 patients (54%) were included in the PD group, and 61 (46%) were in the non-PD group. The baseline patient characteristics are shown in Table 1. There were no significant differences between the two groups except lower triglyceride and higher BNP levels in the PD group compared to the non-PD group (triglyceride: 126 ± 62 and 162 ± 138 mg/dL, *p* = 0.046; BNP: 57 [24–98] and 33 [15–59] pg/mL, *p* = 0.016). The patients’ age, white blood cell counts, and left atrial diameter showed non-significant trends toward higher values in the PD group than in the non-PD group (age: 63.5 ± 10.4 and 60.7 ± 10.8 years, *p* = 0.124; white blood cell counts: 5300 [4700–6800] and 5000 [4400–6200], *p* = 0.072; left atrial diameter: 38.8 ± 5.6 and 37.3 ± 5.0 mm, *p* = 0.118).

**TABLE 1:** Patient characteristics.

	Overall (*n* = 132)	Periodontitis group (*n* = 71)	Non-periodontitis group (*n* = 61)	*p*-value
Age, years	62.2 ± 10.6	63.5 ± 10.4	60.7 ± 10.8	0.124
Female	36 (27.3%)	18 (25.4%)	18 (29.5%)	0.696
BMI, kg/m^2^	23.8 ± 3.9	23.9 ± 4.4	23.6 ± 4.4	0.907
Hypertension	65 (49.2%)	40 (56.3%)	25 (41.0%)	0.084
Diabetes mellitus	13 (9.8%)	7 (9.9%)	6 (9.8%)	1.000
Dyslipidemia	53 (40.2%)	23 (32.4%)	30 (49.2%)	0.053
CKD, stage 3 or more	15 (11.4%)	12 (16.9%)	3 (4.9%)	0.051
Heart failure	6 (4.4%)	2 (2.8%)	3 (4.9%)	0.662
Old myocardial infarction	5 (3.8%)	2 (2.8%)	3 (4.9%)	0.662
Stroke, TIA, or prior thromboembolism	8 (6.1%)	3 (4.2%)	5 (8.2%)	0.470
CHADS_2_ score	1 [0–1]	1 [0–1]	1 [0–1]	0.332
CHA_2_DS_2_-VASc score	2 [1–2]	2 [1–2]	1 [1–2]	0.595
Drinking	83 (62.9%)	48 (67.6%)	35 (57.4%)	0.279
**Smoking**				
Current smokers	22 (16.7%)	11 (15.5%)	11 (18.0%)	0.816
Former smokers	56 (42.4%)	30 (42.3%)	26 (42.6%)	1.000
Never smokers	53 (40.2%)	30 (42.3%)	23 (37.7%)	0.722
**Medication use**				
Warfarin	32 (24.2%)	20 (28.2%)	12 (19.7%)	0.311
DOAC	98 (74.2%)	51 (71.8%)	47 (77.0%)	0.553
Aspirin	9 (6.8%)	5 (7.0%)	4 (6.6%)	1.000
Beta-blocker	34 (25.8%)	16 (22.5%)	18 (29.5%)	0.426
Dihydropyridine CCB	40 (30.3%)	22 (31.0%)	18 (29.5%)	1.000
ACEI/ARB	49 (37.1%)	25 (35.2%)	24 (39.3%)	0.718
Diuretics	10 (7.6%)	6 (8.5%)	4 (6.6%)	0.752
MRA	4 (3.0%)	2 (2.8%)	2 (3.3%)	1.000
Statins	40 (30.3%)	17 (23.9%)	23 (37.7%)	0.092
**Laboratory findings**				
White blood cells, /μL	5200 [4500–6500]	5300 [4700–6800]	5000 [4400–6200]	0.072
Hemoglobin, g/dL	14.0 ± 1.3	14.0 ± 1.4	14.1 ± 1.3	0.798
Albumin, g/dL	4.4 ± 0.3	4.4 ± 0.3	4.4 ± 0.4	0.980
Creatinine, mg/dL	0.82 [0.71–0.95]	0.82 [0.74–0.92]	0.83 [0.70–0.97]	0.803
Total cholesterol, mg/dL	200 ± 33	195 ± 30	205 ± 35	0.083
Triglyceride, mg/dL	143 ± 106	126 ± 62	162 ± 138	0.046
HDL-cholesterol, mg/dL	62 ± 16	62 ± 15	62 ± 17	0.835
LDL-cholesterol, mg/dL	120 ± 29	118 ± 27	122 ± 31	0.362
HbA1c, %	5.8 ± 0.7	5.8 ± 0.7	5.8 ± 0.7	0.693
CRP, mg/dL	0.06 [0.03–0.11]	0.05 [0.03–0.14]	0.06 [0.04–0.08]	0.587
CRP ≥ 0.1 mg/dL	36 (27.3%)	25 (35.2%)	11 (18.0%)	0.032
Brain natriuretic peptide, pg/mL	43.2 [18.0–83.5]	56.7 [24.4–97.5]	32.9 [15.3–59.4]	0.016
**Echocardiographic measures**				
Left ventricular ejection fraction, %	65.4 ± 8.7	65.1 ± 9.6	65.8 ± 7.6	0.686
Left atrial diameter, mm	38.1 ± 5.3	38.8 ± 5.6	37.3 ± 5.0	0.118

BMI, body mass index; CKD, chronic kidney disease; TIA, transient ischemic attack; CHADS_2_ score, congestive heart failure, hypertension, age, diabetes, previous stroke/transient ischemic attack (2 points) score; CHA_2_DS_2_-VASc, congestive heart failure, hypertension, age (>65 = 1 point, >75 = 2 points), diabetes, previous stroke/transient ischemic attack (2 points) vascular disease score; DOAC, direct oral anticoagulant; CCB, calcium channel blockers; ACEI, angiotensin-converting enzyme inhibitor; ARB, angiotensin II receptor blocker; MRA, mineralocorticoid receptor antagonist; HDL, high-density lipoprotein; LDL, low-density lipoprotein; HbA1c, glycated hemoglobin; CRP, C-reactive protein.

### 3.2. Periodontal status

The baseline periodontal conditions in the two groups are summarized in Table 2. Patients in the PD group had significantly greater PPD and CAL values, more prevalent BoP-positive teeth, and higher CPI values in comparison with those in the non-PD group, suggesting the worse periodontal status in the PD group. The antigens of *P. gingivalis* and *P. intermedia* were more frequently detected in the periodontal pocket and in the saliva in the PD group than in the non-PD group. The serum IgG titer for *P. gingivalis* was significantly higher in the PD group than in the non-PD group.

**TABLE 2:** Periodontal conditions.

	Overall (*n* = 132)	Periodontitis group (*n* = 71)	Non-periodontitis group (*n* = 61)	*p*-value
Number of remaining teeth	26 [21–28]	25 [20–28]	26 [22–28]	0.215
Maximum PPD, mm	4.0 [3.0–5.0]	4.0 [4.0–6.5]	3.0 [3.0–3.0]	<0.001
Mean PPD, mm	2.3 [2.1–2.6]	2.6 [2.4–2.9]	2.1 [1.9–2.2]	<0.001
Maximum CAL, mm	5.0 [4.0–6.3]	6.0 [4.0–7.0]	4.0 [3.0–5.0]	<0.001
Mean CAL, mm	2.6 [2.3–3.3]	3.0 [2.7–3.8]	2.3 [2.2–2.6]	<0.001
Positive rate of BoP	0.10 [0.00–0.28]	0.20 [0.08–0.40]	0.00 [0.00–0.08]	<0.001
Maximum CPI	3 [2–3]	3 [3–4]	2 [1–2]	<0.001
**Pathogens in the periodontal pocket**				
*P. gingivalis*	88 (67.7%)	55 (78.6%)	33 (55.0%)	0.005
*P. intermedia*	31 (23.8%)	23 (32.9%)	8 (13.3%)	0.013
*A. actinomycetemcomitans*	20 (15.4%)	10 (14.3%)	10 (16.7%)	0.809
**Pathogens in the saliva**				
*P. gingivalis*	90 (71.4%)	57 (82.6%)	33 (57.9%)	0.003
*P. intermedia*	40 (31.7%)	29 (42.0%)	11 (19.3%)	0.007
*A. actinomycetemcomitans*	28 (22.2%)	14 (20.3%)	14 (24.6%)	0.668
**Serum antibody titer, U/mL**				
*P. gingivalis*	124 [43–399]	210 [71–548]	94 [21–178]	<0.001
*P.intermedia*	332 [192–595]	318 [178–519]	386 [199–600]	0.567
*A. actinomycetemcomitans*	44 [25–91]	45 [25–83]	39 [25–107]	0.886

PPD, probing pocket depth; CAL, clinical attachment level; BoP, bleeding on probing; CPI, Community Periodontal Index; *P. gingivalis, Porphyromonas gingivalis; P. intermedia, Prevotella intermedia; A. actinomycetemcomitans, Aggregatibacter actinomycetemcomitans*.

### 3.3. Catheter ablation procedures

The procedures performed on each patient are shown in Table 3. PV isolation was successfully achieved in all patients using a radiofrequency catheter (81.1%), cryoballoon (15.2%), or hot balloon (3.8%). There were no significant differences in procedure strategies and procedure-related complications between the PD and non-PD groups.

**TABLE 3:** Ablation procedures.

	Overall (*n* = 132)	Periodontitis group (*n* = 71)	Non-periodontitis group (*n* = 61)	*p-*value
**Energy source for catheter ablation**				
Radiofrequency ablation	107 (81.1%)	60 (84.5%)	47 (77.0%)	0.373
Cryoballoon ablation	20 (15.2%)	9 (12.7%)	11 (18.0%)	0.468
Hot balloon ablation	5 (3.8%)	2 (2.8%)	3 (4.9%)	0.662
**Ablation strategy**				
PVI without adjunctive ablation except CTI block	101 (76.5%)	53 (74.6%)	48 (78.7%)	0.682
PVI with adjunctive ablation except CTI block	31 (23.5%)	18 (25.4%)	13 (21.3%)	0.682
CTI block	113 (85.6%)	62 (87.3%)	51 (83.6%)	0.623
**Procedure-related complications**				
Stroke/TIA	0	0	0	
Cardiac tamponade	2	2	0	
Congestive heart failure	2	1	1	
Lower respiratory tract infections	4	2	2	
Phrenic nerve palsy	2	1	1	
Pulmonary vein stenosis	1	1	0	
Pericarditis	1	1	0	
Arteriovenous fistula	1	0	1	
Total	12 (9.1%)	8 (11.3%)	4 (6.6%)	0.383
Class I or III antiarrhythmic drugs on discharge	39 (29.5%)	22 (31.0%)	17 (27.9%)	0.707

PVI, pulmonary vein isolation; CTI, cavotricuspid isthmus; TIA, transient ischemic attack.

### 3.4. Post-ablation outcomes and predictors of recurrent arrhythmia

**FIGURE 1:**
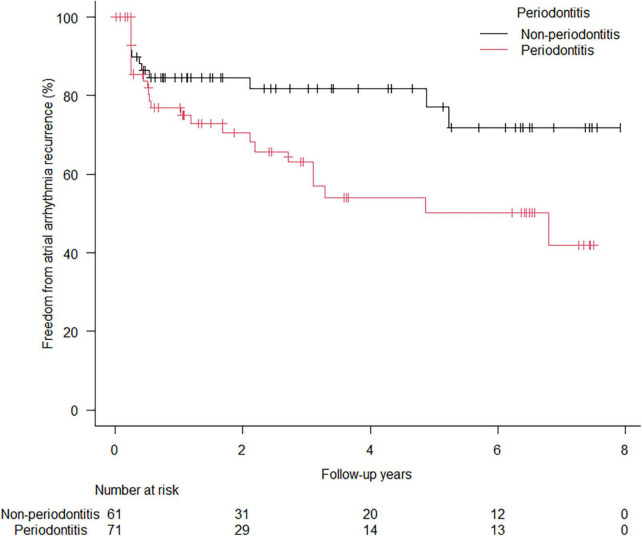
Arrhythmia recurrence-free survival in the patients with and without periodontitis. Kaplan–Meier curve analysis showed worse atrial arrhythmia recurrence-free survival probabilities after the first session of catheter ablation for paroxysmal atrial fibrillation in the patients with periodontitis than in those without periodontitis (64.8% vs. 80.3%, respectively: *p* = 0.024).

**FIGURE 2:**
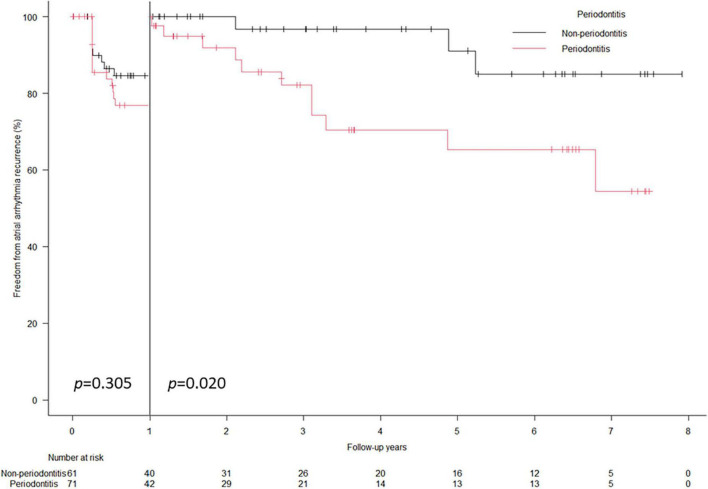
Landmark analysis of arrhythmia recurrence-free survival rate. Landmark analysis showed the cumulative atrial arrhythmia recurrence-free survival probabilities was comparable between PD and non-PD groups during the first 1 year (76.8% vs. 84.6%, log-rank: *p* = 0.305). After 1 year, a significantly worse atrial arrhythmia recurrence-free survival probabilities were demonstrated in the PD group (log-rank: *p* = 0.020).

A total of 37 (28.0%) arrhythmia recurrences were observed in 132 patients at a median follow-up period of 3.0 (1.1–6.4) years. In the Kaplan–Meier analysis, worse atrial arrhythmia recurrence-free survival rate was observed in the PD group than in the non-PD group (64.8% vs. 80.3%, Log-rank: *p* = 0.024) (Figure 1). In the univariate Cox regression analysis, only PD was significantly associated with recurrence (Table 4), while mildly elevated CRP ≥ 0.1 mg/dL showed worse recurrent-free survival rate as compared with those without mildly elevated CRP in Log-rank test (*p* = 0.048) (Supplementary Figure 1), which was not statistically significant in Cox regression analysis (Table 4). The multivariate Cox regression analysis revealed that PD was a significant predictor of arrhythmia recurrence (hazard ratio: 2.063, 95% confidence interval: 1.018–4.182) after adjusting for the age and gender (Table 4). In the landmark analysis, during the initial 1 year, the atrial arrhythmia recurrence-free survival rate was not significantly different between PD and non-PD groups (76.8% vs. 84.6%, Log-rank: *p* = 0.305). After 1 year, there was a continuous separation of the curves in the Kaplan–Meier analysis, with a significantly worse atrial arrhythmia recurrence-free survival rate in the PD group (Log-rank: *p* = 0.020) (Figure 2). The multivariate Cox regression analysis also revealed PD as a significant predictor of arrhythmia recurrence after 1 year (hazard ratio: 5.677, 95% confidence interval: 1.482–21.74) after adjusting for the age and gender.

**TABLE 4:** Predictors of recurrent arrhythmia.

	Univariate Cox regression			Multivariate Cox regression		
	HR	95% CI	*p*-value	HR	95% CI	*p*-value
Age (per year)	1.014	0.980–1.049	0.425	1.008	0.973–1.044	0.663
Male gender	1.092	0.528–2.260	0.812	1.065	0.499–2.274	0.870
BMI (per kg/m^2^)	0.956	0.877–1.042	0.309			
HTN	1.178	0.617–2.248	0.620			
DM	0.469	0.113–1.954	0.299			
DLP	0.835	0.429–1.623	0.594			
CKD	1.206	0.468–3.109	0.699			
LVEF (per%)	1.014	0.975–1.056	0.485			
LA diameter (per mm)	0.990	0.932–1.051	0.732			
CRP (per mg/dL)	1.512	0.562–4.068	0.413			
CRP ≥ 0.1 mg/dL	1.889	0.979–3.644	0.057			
BNP (per pg/mL)	1.001	0.999–1.003	0.337			
Periodontitis	2.135	1.072–4.252	0.031	2.063	1.018–4.182	0.045

HR, hazard ratio; CI, confidence interval; BMI, body mass index; CHF, congestive heart failure; HTN, hypertension; DM, diabetes mellitus; DLP, dyslipidemia; CKD, chronic kidney disease; LVEF, left ventricular ejection fraction; LA, left atrium; CRP, C-reactive protein; BNP, brain natriuretic peptide.

## 4. Discussion

To the best of our knowledge, this is the first study which demonstrates a significant association between PD diagnosed by performing periodontal examinations and the recurrence of arrhythmia after CA. The main finding of this study was that concurrent PD was an independent predictor of recurrent atrial arrhythmia after the first CA for PAF in multivariable Cox regression analysis adjusting for other clinical factors.

### 4.1. PD and systemic inflammation

Periodontitis is a common and well-known chronic oral inflammatory disease. Previous studies reported that subjects with PD are more susceptible than those without PD to the infiltration of periodontal pathogens into the systemic circulation, which potentially causes bacteremia through oral interventions, such as dental treatment or tooth extractions, and even through activities in daily lives, including chewing, biting, toothbrushing, and flossing (12, 13). It is hypothesized that repeated short-time bacteremia activates inflammatory cells, endothelial cells, and other types of cells, leading to the production of systemic inflammatory mediators (14). This is supported by the evidence of elevated plasma CRP (15) and serum interleukin (IL)-6 levels, and lower IL-4 and IL-18 (16) levels in patients with PD compared to healthy controls. Present study, due to a limited sample size, did not show a significant difference in the numerical value of CRP levels between PD and non-PD groups. Nevertheless, the prevalence of mildly elevated CRP levels of ≥0.1 mg/dL was significantly higher in PD group than in non-PD group (Table 1).

### 4.2. Systemic inflammation and AF

A variety of factors, such as the age, other structural heart diseases, the blood pressure, the alcohol intake, obesity, and genetic factors, have been reported to play a role in modifying the electrophysiological substrate of AF (17, 18). Systemic inflammation has also been suggested as one of the modifying factors of AF, which was corroborated by the increased inflammatory markers in the patients with AF (9, 19–22). A case–control study reported that the circulating CRP level was higher in patients with AF than in those without AF (9). A population-based study revealed that the serum CRP level was independently associated with the new occurrence of AF as well as baseline AF (20). Additionally, AF is associated with elevated pro-inflammatory IL-6 levels (19, 21, 22), and left atrial diameters were positively associated with CRP and IL-6 levels, which suggested that the inflammation might promote atrial remodeling (23). A basic research has suggested the interaction between the release of inflammatory cytokines, such as tumor necrosis factor-alpha and IL-6, and the remodeling of the atrial muscle, leading to the development of AF substrates (24). In the present study, although a numerical value of CRP level was not correlated with AF recurrence in Cox proportional hazard models, the patients with mildly elevated CRP ≥ 0.1 mg/dL showed a higher recurrence rate than the counterpart (CRP < 0.1 mg/dL) (Supplementary Figure 1).

### 4.3. PD and AF

In animal models, induced PD led to an inflammatory response and remodeling of the atrial myocardium, facilitating AF inducibility (25). In addition, previous studies have reported the presence of periodontal pathogens in human atheromatous lesions and the association of those pathogens with myocardial damage (26, 27), which suggested that periodontal pathogens may infiltrate into arterial wall or myocardium. Thus, the direct infiltration of periodontal bacteria also may be a potential mechanism of atrial remodeling and predisposition to AF. A Taiwanese nationwide population-based cohort study showed an association between PD and the future development of AF or atrial flutter (6). PD was associated with future arrhythmic events, including AF, atrial tachycardia, and atrial premature beat, and thromboembolic events during the long-term follow-up of the patients with AF (10). Nevertheless, the association of PD with arrhythmia recurrence after CA has not been elucidated. A recent study has shown the association between serum antibody levels to one of the periodontal pathogens and the recurrence of AF (11). This study is the first to demonstrate the association between actual oral health conditions examined in detail by periodontists or dentists and arrhythmia recurrence after CA for PAF, which supports the results of previous studies (6, 10, 11). An interesting finding of this study is the recurrence-free rate curves in the Kaplan–Meier analysis of the PD and non-PD groups separated in the long-term (after 1 year) rather than in short-term (Figures 1, 2). A recent study reported that electrical left atrial remodeling and non-PV triggers were more common in long-term recurrence of AF than in short-term recurrence, whereas PV reconnections were dominant in short-term recurrence (28). In other words, short-term recurrence is mainly associated with procedure-related factors while long-term recurrence is more affected by atrial remodeling, which may develop slowly. This may explain how PD and subsequent systemic inflammation may have an impact on long-term recurrence through atrial remodeling rather than on short-term outcomes. Although the present study could not show the association of PD with the evidence of atrial remodeling such as enlarged atrial dimension or greater atrial low voltage area because of the small sample size and lack of 3D imaging data or voltage map, future prospective studies may address the impact of PD on electrophysiological findings in AF.

### 4.4. Clinical implications

Periodontitis is a common disease worldwide, and at the same time, it is preventable and modifiable with oral health care (29, 30). Previous meta-analyses revealed, with a high sensitivity, that CRP was significantly reduced after periodontal intervention (31). Additionally, a previous randomized study showed a positive effect of the intensive treatment of PD on decreasing serum proinflammatory cytokine levels, including IL-6 (32). Furthermore, the impact of dental scaling on the significant reduction of new-onset AF has also been reported (33). Considering these results, it might be reasonable to posit that dental intervention to maintain periodontal health may reduce AF recurrence after CA. Further prospective studies are required to elucidate the impact of periodontal health care on the outcomes of CA.

### 4.5. Limitations

There are several limitations in this study. First, this is a single-center observational study. Although consecutive patients were enrolled in the study, potential selection bias cannot be ruled out. Further studies are required to prove a causal relationship between PD and AF. Second, this study comprised relatively small number of patients which precluded the assessment of the impact of each component of periodontal status on the arrhythmia recurrence after CA. In addition, because of limited sample size, it was difficult to determine the interaction and collinearity of PD with other clinical factors related with arrhythmia recurrence. Likewise, relatively small number of cases in our study may not have had sufficient statistical power to demonstrate significant associations of known risk factors with AF recurrence. Future studies will have to prospectively explore collinearity and interaction of PD and known predictors of recurrent AF with sufficient sample size. Third, arrhythmia recurrence after CA was diagnosed based on electrocardiogram documentation or recording of a 24-h Holter or a 1-week event loop recorder. Thus, asymptomatic recurrence may have been missed by examinations, and the recurrence rate might have been underestimated. Fourth, changes in the periodontal status before and after index CA were not assessed, and the information on the periodontal treatment was also lacking, which may have affected the clinical outcomes. Fifth, strategies in CA procedures were left to the operators’ discretion, which may have affected the outcomes. Finally, this study excluded patients with persistent AF because of a different recurrence rate from PAF.

## 5. Conclusion

In this study, we investigated the patients who underwent periodontal examinations during admission for fist CA due to PAF. The presence of PD diagnosed by periodontists at baseline was a significant risk factor for recurrent atrial arrhythmia in long-term follow-up. Future studies are required to confirm whether oral health care may reduce the recurrence of PAF after CA.

## Data availability statement

The raw data supporting the conclusions of this article will be made available by the authors, without undue reservation.

## Ethics statement

The studies involving human participants were reviewed and approved by the IRB of the Tokyo Medical and Dental University. The patients/participants provided their written informed consent to participate in this study.

## Author contributions

AT was responsible for the data collection, investigation, analysis, and original draft writing. TY participated in the conceptualization and methodology of the work and edited the manuscript. NA, YS-W, and TN contributed to the data collection and investigation. SM, YM, MG, MI, and TI reviewed and edited the manuscript. TS supervised the work. All authors listed have made a substantial contribution to the work and approved the final version of the manuscript.

## Supplementary material

Supplementary Figure 1 Arrhythmia recurrence-free survival according to the C-reactive protein levels. Kaplan–Meier curve analysis showed worse post-ablation atrial arrhythmia recurrence-free survival probabilities in the patients with CRP ≥ 0.1 mg/dL than those with CRP < 0.1 mg/dL (log-rank: *p* = 0.048).
