# Supplementary figures and images for: Periodontitis was associated with worse clinical outcomes after catheter ablation for paroxysmal atrial fibrillation
Source: Front Cardiovasc Med. 2023 Jan 9;9:1061243. doi: 10.3389/fcvm.2022.1061243 (PMC9868319; doi:10.3389/fcvm.2022.1061243)

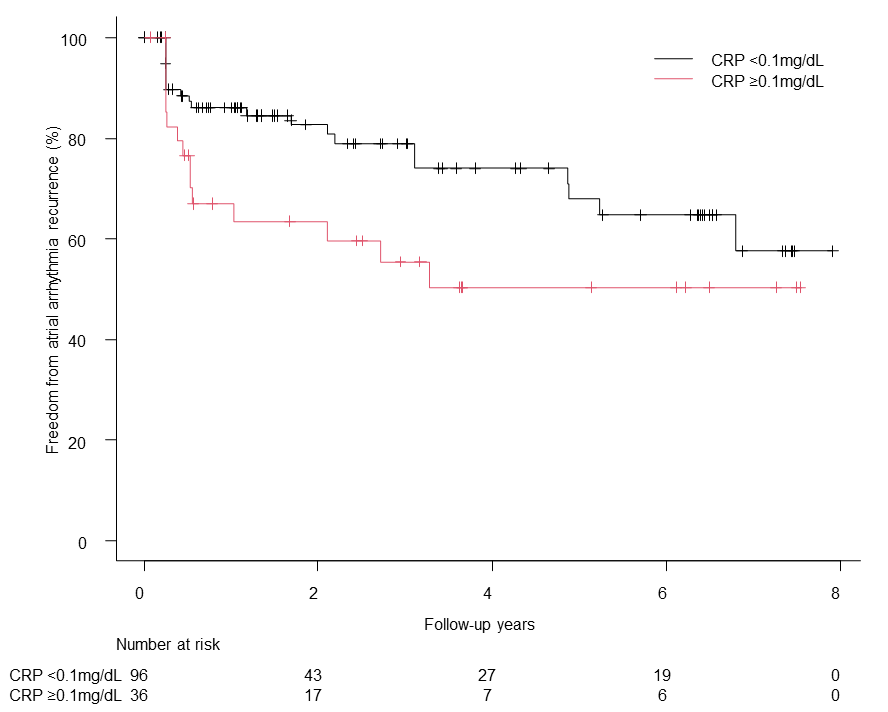

Supplement: Supplementary Figure 1 — Arrhythmia recurrence-free survival according to the C-reactive protein levels. Kaplan–Meier curve analysis showed worse post-ablation atrial arrhythmia recurrence-free survival probabilities in the patients with CRP ≥ 0.1 mg/dL than those with CRP < 0.1 mg/dL (log-rank: p = 0.048). [file Image_1.TIF]
